# Novel HIV-1 Recombinants Spreading across Multiple Risk Groups in the United Kingdom: The Identification and Phylogeography of Circulating Recombinant Form (CRF) 50_A1D

**DOI:** 10.1371/journal.pone.0083337

**Published:** 2014-01-15

**Authors:** Geraldine M. Foster, John C. Ambrose, Stéphane Hué, Valerie C. Delpech, Esther Fearnhill, Ana B. Abecasis, Andrew J. Leigh Brown, Anna Maria Geretti, on behalf of the UK HIV Drug Resistance Database

**Affiliations:** 1 University of Liverpool, Liverpool, United Kingdom; 2 University College London, London, United Kingdom; 3 Public Health England, London, United Kingdom; 4 MRC Clinical Trials Unit, London, United Kingdom; 5 Universidade Nova de Lisboa, Lisbon, Portugal; 6 University of Edinburgh, Edinburgh, United Kingdom; Institut Pasteur of Shanghai, Chinese Academy of Sciences, China

## Abstract

**Background:**

An increase in non-B HIV-1 infections among men who have sex with men (MSM) in the United Kingdom (UK) has created opportunities for novel recombinants to arise and become established. We used molecular mapping to characterize the importance of such recombinants to the UK HIV epidemic, in order to gain insights into transmission dynamics that can inform control strategies.

**Methods and Results:**

A total of 55,556 *pol* (reverse transcriptase and protease) sequences in the UK HIV Drug Resistance Database were analyzed using Subtype Classification Using Evolutionary Algorithms (SCUEAL). Overall 72 patients shared the same A1/D recombination breakpoint in *pol*, comprising predominantly MSM but also heterosexuals and injecting drug users (IDUs). In six MSM, full-length single genome amplification of plasma HIV-1 RNA was performed in order to characterize the A1/D recombinant. Subtypes and recombination breakpoints were identified using sliding window and jumping profile hidden markov model approaches. Global maximum likelihood trees of *gag*, *pol* and *env* genes were drawn using FastTree version 2.1. Five of the six strains showed the same novel A1/D recombinant (8 breakpoints), which has been classified as CRF50_A1D. The sixth strain showed a complex CRF50_A1D/B/U structure. Divergence dates and phylogeographic inferences were determined using Bayesian Evolutionary Analysis using Sampling Trees (BEAST). This estimated that CRF50_A1D emerged in the UK around 1992 in MSM, with subsequent transmissions to heterosexuals and IDUs. Analysis of CRF50_A1D/B/U demonstrated that around the year 2000 CRF50_A1D underwent recombination with a subtype B strain.

**Conclusions:**

We report the identification of CRF50_A1D, a novel circulating recombinant that emerged in UK MSM around 1992, with subsequent onward transmission to heterosexuals and IDUs, and more recent recombination with subtype B. These findings highlight the changing dynamics of HIV transmission in the UK and the converging of the two previously distinct MSM and heterosexual epidemics.

## Introduction

The dynamics of the HIV epidemic are changing in the United Kingdom (UK). By the end of 2010, approximately 91,500 people were estimated to be living with HIV, including 40,100 men who have sex with men (MSM), 47,000 heterosexual men and women and 2,300 injecting drug users (IDUs) [1]. In previous years, heterosexual infections, which were mostly imported, had overtaken infections in MSM, 81% of which are indigenously acquired. However, the trend has now reversed, reflecting a decline in the number of infections acquired abroad, and a corresponding increase in the number of infections acquired in the UK [1]. These changes have considerable potential to modify established epidemic patterns.

Mapping the molecular epidemiology of HIV infection can provide valuable insights into transmission networks and thereby inform prevention and containment strategies.

As seen in other Western countries, including Italy and the United States [2], [3], in the UK the HIV epidemic among MSM was traditionally composed of nearly uniformly subtype B infections, contrasting with the variety of non-B subtypes found in heterosexual infections, which are predominantly imported from sub-Saharan Africa [4], [5]. Non-B infections have been increasing in recent years in MSM [2], [6], [7]. Under favorable conditions such as those found in populations at risk of multiple HIV exposures, novel recombinant strains can emerge and become established, supplanting previous patterns of infection. The emergence of recombinant HIV-1 strains among UK MSM was proposed by Gifford *et al.* in 2007, based upon the detection of a potentially novel subtype A recombinant in phylogenetic analyses of *pol* gene sequences [6].

The aim of this study was to seek firmer evidence that novel recombinant forms of HIV are emerging in the UK MSM population. Through the screening of a large national database containing reverse transcriptase and protease sequences from patients undergoing drug resistance testing in routine care, and subsequent near full-length, single genome sequencing (SGS) of clinical isolates, we identified a novel A1/D circulating recombinant form, now registered as CRF50_A1D, which first emerged in the UK around 1992. We show that over time, CRF50_A1D spread geographically and entered heterosexual and IDU transmission networks, followed by recombination with a subtype B strain and emergence of the unique recombinant form (URF) CRF50/B/U. These findings indicate that the two previously distinct HIV epidemics in MSM and heterosexuals have started to converge in the UK, creating opportunities for greater HIV genetic diversification.

## Methods

### Study population

The UK HIV Drug Resistance Database (HIV-DRD) (http://www.hivrdb.org.uk/) and Public Health England (previously the Health Protection Agency) provided access to *pol* sequences and demographic and clinical data. The HIV-DRD is a national repository of protease and reverse transcriptase sequences obtained by Sanger sequencing in patients undergoing drug resistance testing in routine care. At the time of the analysis, there were 55,556 sequences in the database from both antiretroviral treatment (ART)-naïve and ART-experienced patients. Based upon data from Gifford et al. indicating that a potentially novel subtype A (sub-subtype A1) recombinant was circulating among MSM in the UK [6], sequences from patients infected with sub-subtype A1 were selected from the database for further analysis. The REGA Subtyping tool and bootscanning analysis using Simplot v3.5.1 were used to subtype recombinant sequences [8]. Subsequent subtyping was performed using Subtype Classification Using Evolutionary Algorithms (SCUEAL) [9]. Stored plasma samples from six selected patients were retrieved for further sequence analysis.

### Ethics statement

The Ethics Committee of the Royal Free Hospital in London approved the anonymized use of stored plasma samples collected during routine care. Personnel from the HIV-DRD selected patients with different identifiers that attended centers with more than 1000 patients in follow-up and were not known to be related, and communicated the HIV-DRD identifier to the center of care to allow sample retrieval from storage. Samples were anonymized prior to shipment to the laboratory for sequencing.

### Near full-length single genome sequencing

Near full-length SGS was performed using a protocol adapted from the Centre for HIV/AIDS Vaccine Immunology (CHAVI-MBSC 2009, unpublished) and optimised for plasma specimens with low HIV-1 RNA levels. Briefly, 140 µl of plasma was adjusted through either dilution or centrifugation to contain 20,000 HIV-1 RNA copies. RNA was extracted using the QiAmp Viral RNA Mini kit (Qiagen, Hilden, Germany) according to the manufacturer's instructions, with a final elution volume of 65 µl. All extracted RNA was immediately transcribed with Superscript III First Strand Synthesis Supermix (Life Technologies, Paisley, UK) using the following protocol per reaction: 0.25 µM of reverse primer 1.R3.B3R 5′-ACTACTTGAAGCACTCAAGGCAAGCTTTATTG (CHAVI-MBSC 2009, unpublished), 1.87 µl nuclease free water, 2.5 µl annealing buffer and 15 µl (5,000 copies) RNA template were denatured at 65°C for 5 minutes. Reactions were placed on ice for at least 1 minute before adding 25 µl of reaction mixture and 5 µl of enzyme mixture. Reverse transcription was performed at 50°C 90 minutes, 55°C 90 minutes, 85°C 5 minutes. An extra 2 µl of Superscript III RT Enzyme was added prior to increasing the temperature to 55°C.

A two-step protocol was used to ensure single genome amplification. The first step was a limiting dilution to assess that cDNA was amplifying at rates suggesting near-ideal extraction and reverse transcription conditions, i.e. that the undiluted cDNA contained 100 copies/µl. Following this, cDNA was amplified using a 1∶200 cDNA dilution (a theoretical input of 1 copy/reaction). In circumstances where the extracted number of HIV-1 RNA copies was below 20,000 copies, or the limiting dilution plate suggested that the sample was amplifying suboptimally, dilutions were adjusted accordingly.

Both first round and nested PCR reactions used the Platinum PCR Supermix High Fidelity Kit (Life Technologies, Paisley, UK). First round reactions comprised 45 µl PCR supermix, 0.25 µM each forward primer 1.U5.B1F 5′CCTTGAGTGCTTCAAGTAGTGTGTGCCCGTCTGT and reverse primer 1.R3.B3R, 0.5 µl nuclease free water and 2 µl cDNA. Cycling conditions were 94°C 2 minutes, followed by 40 cycles of 94°C 15 s, 60°C 30 s, 68°C 9.5 m, and a final extension at 68°C 20 minutes. Nested PCR reactions were identical to the first round reactions, excepting the use of forward primer 2.U5.B4F 5′-AGTAGTGTGTGCCCGTCTGTTGTGTGACTC, reverse primer 2.R3.B6R 5′-TGAAGCACTCAAGGCAAGCTTTATTGAGGC, and 45 cycles of PCR. The resulting 9 kb product spanned HXB2 nucleotides 552–9636.

Positive nested PCR reactions were identified using 1% agarose gel electrophoresis. Filtered PCR products were directly sequenced using fluorescently labeled dideoxy chain terminators (BigDye Terminator v3.1 Cycle Sequencing Assay, Life Technologies) and an automated ABI 3730×l sequencer. Sequencing primers were either sourced from the in-house protocols of the Molecular Biology and Sequencing Core at the Centre for HIV/AIDS Vaccine Immunology or protocols available in published literature [10]–[12], or designed using Primer3 version 4.0 (http://frodo.wi.mit.edu/primer3) (Supplementary information 1). Sequencing reactions were repeated until near full bi-directional coverage was obtained, and sequences were assembled using SeqScape version 2.6 software (Life Technologies). Fragment sequences from individual sequencing primers were examined for mixed bases; where evidence of amplification of >1 target molecule was found, amplification and sequencing was repeated.

### Phylogenetic and recombination analyses

Recombination analyses and subtype assignation was performed using the Recombinant Identification Program (RIP) (http://www.hiv.lanl.gov/content/sequence/RIP/RIP.html), jpHMM (http://jphmm.gobics.de) and Simplot [13]. For RIP and Simplot analyses, a window size of 400 bp and a step size of 20 were used. Sequences were gap-stripped and genetic distances were calculated using Kimura 2-p parameters. Simplot analyses were performed using a full-length reference alignment of 78 pure subtype sequences. Bootscanning of the query sequence was performed using subtypes A1, B, D, and F2 with informative sites analysis. Recombination breakpoints were set using the highest statistically significant X^2^ value around the 50% crossover point between subtypes. The statistical significance of the identified breakpoints was assessed using Fisher's exact test. Following breakpoint assignment, slices of the alignment corresponding to putative pure subtype regions between each breakpoint were created and saved for downstream analyses. Likelihood mapping of each slice was used to assess phylogenetic signal prior to maximum likelihood analysis and was performed using TreePuzzle [14].

Likelihood parameters for each putatively pure subtype region of the HIV genome were estimated using PAUP version 4.0 (Sinauer Associates, Massachusetts, USA). Maximum likelihood analysis was performed using the PhyML implementation housed at the ATGC server (http://www.atgc-montpellier.fr/phyml/). 1000 bootstrapping replicates were performed, with the exception of alignment slices 5 and 8, which were restricted to 100 replicates to limit computational requirements.

Phylogenetic trees were visualized using Dendroscope version 2.3 (available from http://ab.inf.uni-tuebingen.de/data/software/dendroscope3/download/welcome.html) and FigTree v1.3.1 (http://tree.bio.ed.ac.uk/software/figtree/). Schematics of finalized recombinant structures were drawn using the Recombinant HIV-1 Drawing Tool (RDT), available from the Los Alamos website (http://www.hiv.lanl.gov/content/sequence/DRAW_CRF/recom_mapper.html).

Beyond full-length sequencing, further instances of CRF50_A1D infections were identified using BLAST to compare three representative CRF50_A1D sequences (33365, 8179, 40534) to the sequences contained in the HIV-DRD. The top 500 hits for each sequence were analyzed for recombination profiles and breakpoints using jpHMM and SCUEAL. Sequences with identical subtype classifications and with a jpHMM breakpoint that fell within the SCUEAL 95% confidence interval were considered CRF50_A1D matches for further investigation.

The likely global origin of the parental subtype A1 and D strains of CRF50 was investigated using global subtype alignments containing subtype A1 or D sequences from every country in the Los Alamos National HIV Database with a greater than 10% representation of either subtype. These sequences were selected by geographical region only and no further data was sought. One alignment for each subtype was generated for partial *gag*, *pol*, and *env* genes. The *pol* gene trees were supplemented with pure subtype A and D sequences from the HIV-DRD. Approximate maximum likelihood analysis was performed using FastTree 2.1 using a GTR+CAT model (http://meta.microbesonline.org/fasttree/).

The emergence and distribution of identified CRF50_A1D sequences in the UK was analyzed using time-scaled analyses implemented in BEAST. The 72 putative CRF50_A1D sequences were aligned with 8 reference A1 and D sequences from East Africa, the 4 closest sequence matches in the NCBI database, and 4 subtype C sequences as an outgroup. A total of 3× MCMC runs of 1×10^8^ states were performed and combined for each analysis. The GTR+Γ nucleotide model was used with a relaxed, log-normal molecular clock and a Bayesian skyline coalescent with a constant population distribution and 10 skyline groups. For discrete phylogeographic analyses phylogeographic operators as detailed in (http://beast.bio.ed.ac.uk/Discrete_Phylogeographic_Analysis) were used with a resampled time-scaled tree as input. In order to preserve patient anonymity, the locations of individual clinics were not used as inputs into the phylogeographic analysis. Instead, the geographic location for patients was determined using aggregated center data which groups clinics together in approximate locations; the central latitude/longitude point of each aggregate was used as patient location. Following BEAST analysis, phylogeographic trees were converted to .kml format and visualized in Google Earth.

The A1/D recombinant structure was registered with the Los Alamos National Database as CRF50_A1D. All six full-length sequences were submitted to Genbank (accession numbers: JN417236-JN417241); the reference sequence for CRF50_A1D is JN417236.

## Results

### Identification and amplification of putative novel recombinant sequences

Following screening of 55,556 HIV-1 *pol* gene sequences in the HIV-DRD, sequences from eight subjects were identified that appeared to share a novel recombinant structure. Stored plasma samples from six of these eight subjects were retrieved from three centers in the UK. The samples had been collected between 2000 and 2011 and stored at −80°C under routine conditions. The HIV-1 RNA load measured at the time of sample collection ranged from 9,148 to 500,000 copies/ml and the available sample volumes ranged from 270 to 1500 µl. The optimal 20,000 HIV-1 RNA copies for sequencing were recovered from three of the six samples; all six specimens, however, were successfully amplified at lower than the 30% Poisson distribution set-point for single genome amplification following limiting dilution.

### Recombination analyses

**Figure 1:**
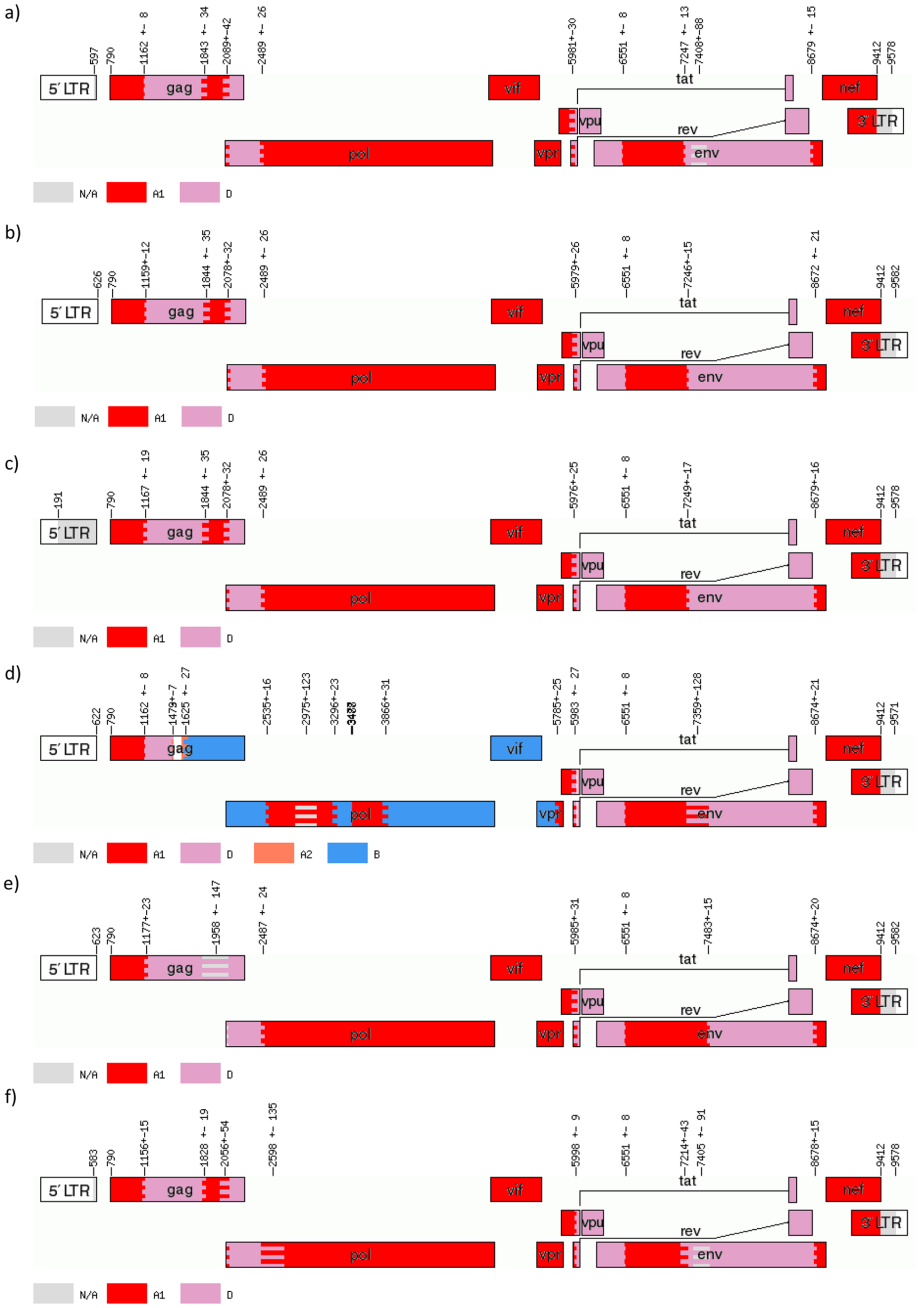
jpHMM analysis of six recombinant HIV-1 sequences. Putative recombinant HIV-1 sequences were submitted to the online implementation of jpHMM at the GOBICS server. The program used its own stored reference alignment and statistical algorithm to determine subtype classifications, breakpoint locations and 95% confidence intervals. Breakpoint locations and confidence intervals are marked on each plot and are equivalent to HXB2 numbering. In each plot, subtype A1 is represented in red, subtype A2 in coral (plot d only), subtype D in lavender, and subtype B in blue (plot d only). Areas of subtype uncertainty are grey. Five specimens (a, b, c, e, and f) showed largely identical A1/D structures, whereas one specimen (d) showed a complex A1/A2/D/B/U structure. a) Specimen 33365; b) Specimen 8179; c) Specimen 40534; d) Specimen 34567; e) Specimen 11762; f) Specimen 12792.

**Figure 2:**
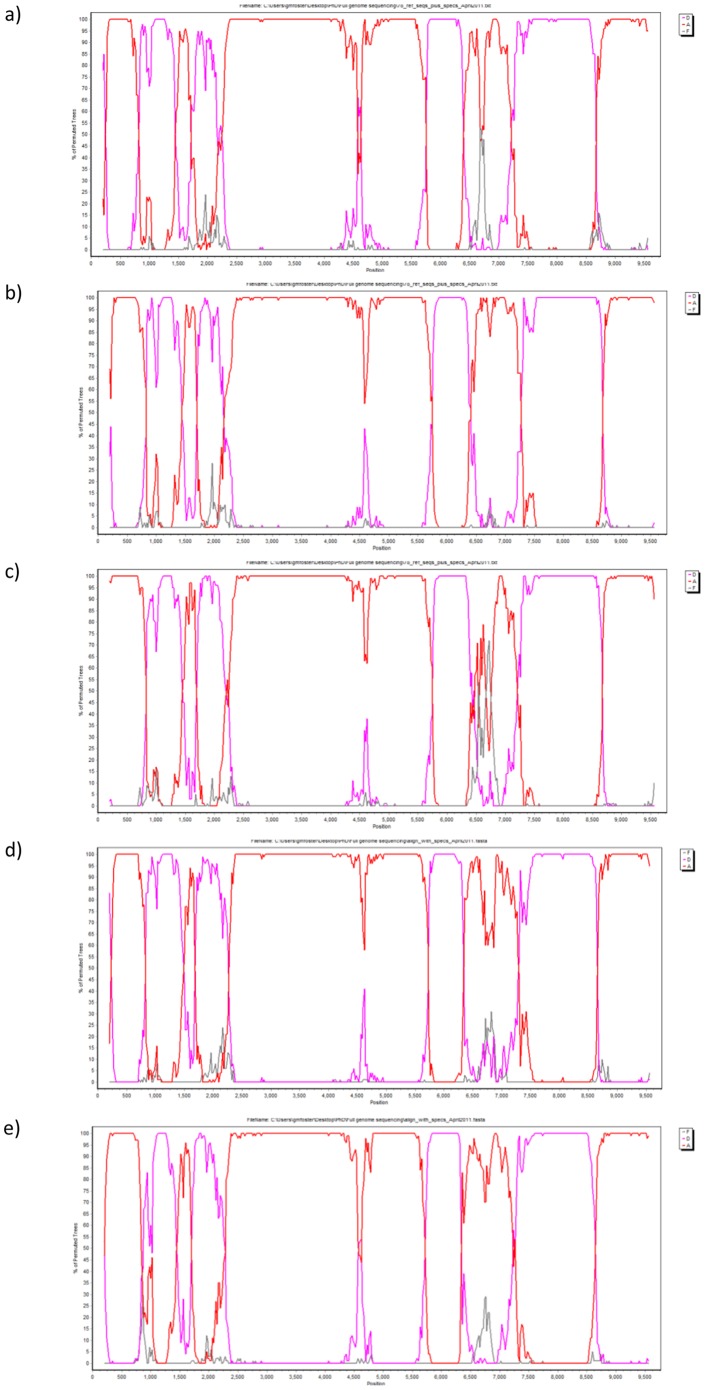
Bootscanning plots for five A1/D recombinants. Bootscanning plots from Simplot sliding window analysis using a window size of 400(not HXB2 numbering). Subtype A is represented in red, subtype D in lavender, and subtype F (outgroup) in grey. All five specimens (33365, 8179, 40534, 11762, 12792) showed identical bootscanning plots, with five subtype A1 regions and four subtype D regions. A) Specimen 33365; b) Specimen 8179; c) Specimen 40534; d) Specimen 11762; e) Specimen 12792.

RIP analysis of six specimens showed a putatively identical A1/D structure with five of the six clinical isolates analyzed (33365, 8179, 40534, 11762, 12792); the sixth isolate (34567) showed a complex A1/B/D structure (data not shown). jpHMM analysis similarly identified five isolates with largely identical A1/D structures (33365, 8179, 40543, 11762, 12792) and one isolate with a complex A1/A2/D/B/U structure (34567) (Figure 1). The breakpoint locations for the five A1/D specimens are summarized in Table 1. Generally, the jpHMM breakpoint locations and subtype classifications showed a good level of consistency among the five A1/D specimens, and with the structure suggested by the RIP screening. Two potential structural discrepancies were suggested by jpHMM. With specimen 11762, the p2–p7 regions of *gag* showed a lower degree of subtype A1 identity than observed with the other four A1/D specimens; however, overlapping confidence intervals indicated that the uncertainty was unlikely to reflect a true structural difference. With specimens 33365 and 12792, two regions of *env* were designated as subtype D/uncertain in the jpHMM plots; however bootscanning of these regions confirmed the subtype D classification (Figure 2).

**Table 1:** jpHMM-assigned breakpoint locations (with 95% confidence intervals) for five HIV-1 A1/D recombinant sequences^a^.

Break point	Study number					Gene	Region
	33365	8179	40534	11762	12792		
1	1162 (1154–1170)	1159 (1147–1171)	1167 (1148–1186)	1177 (1154–1200)	1156 (1141–1171)	*gag*	p24
2	1843 (1809–1877)	1844 (1809–1879)	1844 (1809–1879)	1958* (1811–2105)	1828 (1809–1847)	*gag*	p24
3	2089 (2047–2131)	2078 (2046–2110)	2078 (2046–2110)	2078 (2046–2110)	2056 (2002–2110)	*gag*	p1
4	2489 (2463–2515)	2489 (2465–2515)	2489 (2465–2515)	2487 (2475–2499)	2598† (2463–2733)	*pol*	PR
5	5981 (5951–6011)	5979 (5953–6005)	5976 (5951–6001)	5985 (5973–5997)	5998 (5989–6007)	*tat/rev*	
6	6551 (6543–6559)	6551 (6543–6559)	6551 (6543–6559)	6551 (6539–6563)	6551 (6543–6559)	*env*	gp120
7	7247 (7234–7260)	7246 (7231–7261)	7249 (7232–7266)	7483 (7471–7495)	7214 (7171–7257)	*env*	gp120
8	8679 (8664–8694)	8672 (8651–8693)	8679 (8663–8695)	8674 (8662–8686)	8678 (8663–8693)	*env*	gp41

^a^ Breakpoint locations as determined by jpHMM with HXB2 numbering. The breakpoint locations are generally consistent across the five specimens, indicating that the same A1/D recombinant structure is shared.

This corresponds to a region of subtype D uncertainty;

This corresponds to a region of subtype A uncertainty; refer to Figure 1. PR = Protease.

Breakpoints identified using bootscanning and informative sites analyses were consistent with those identified using jpHMM. All five A1/D specimens (33365, 8179, 40534, 11762, 12792) showed identical bootscanning plots, with five subtype A1 regions and four subtype D regions.

The jpHMM analysis of specimen 34567 further clarified the recombinant structure of this complex isolate. Two clear regions with the same structure as the five A1/D specimens were identified, at the very beginning of *gag*, which had an identical A1/D breakpoint (1162±8), and from the breakpoint in *tat/rev* (5983±23) to the end of the genome. This suggested that this specimen resulted from a further recombination event between the A1/D recombinant and a subtype B strain.

### Maximum likelihood analyses

**Figure 3:**
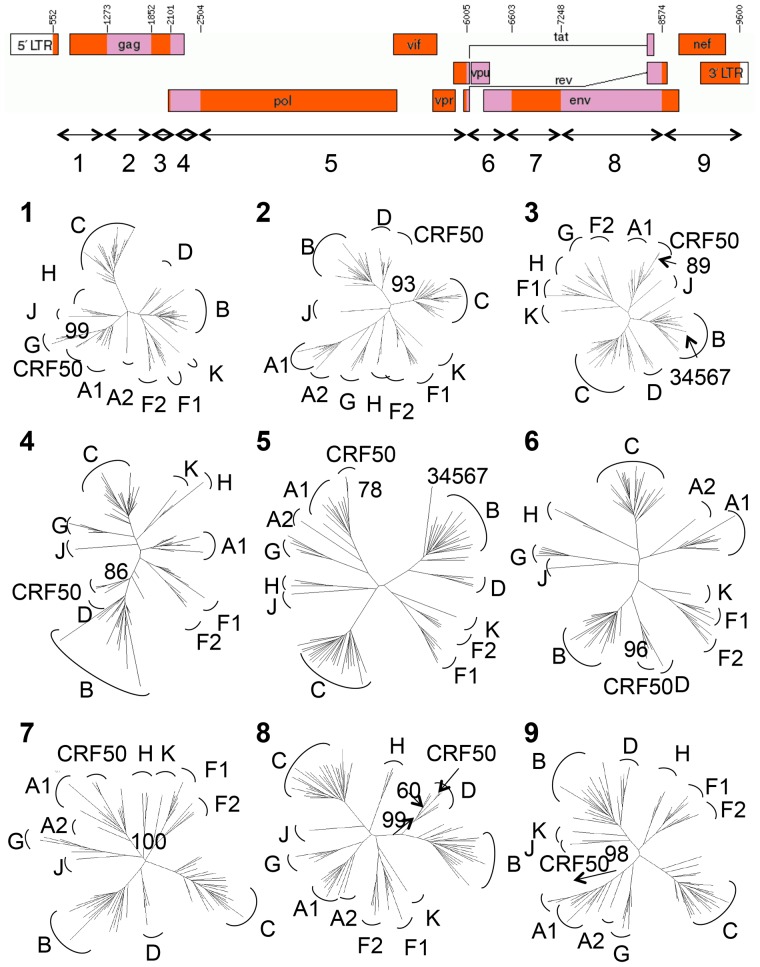
Recombinant map of CRF50_A1D and maximum likelihood phylogenetic trees of non-recombinant fragments. Maximum likelihood trees of putative non-recombinant fragments from specimens 33365, 8179, 40534 and 34567 drawn using PhyML with PAUP-defined parameters. HIV-1 subtypes used for analysis were A–D, F, G, H, J, K. Numbers indicate bootstrapping support from 1000 replicates (excepting slice 5; 100 replicates). 70% bootstrap support and monophyletic clustering were the criteria for subtype classification. The recombinant map was drawn using the RDT program at Los Alamos. Component subtype fragments are labeled 1–9 on the genome map, corresponding with numbered phylogenetic trees. The genomic regions in which the URF specimen 34567 did not cluster with the CRF50_A1D specimens are indicated in the appropriate trees.

Maximum likelihood trees of putative non-recombinant fragments drawn using PhyML with PAUP-defined parameters showed that each fragment of each specimen clustered with the pure subtype (A1 or D) indicated by the bootscanning analysis. Results obtained with A1/D specimens 33365, 8179, and 40534, and complex specimen 34567 are shown in Figure 3. The A1/D structure was predominantly subtype A1 in *pol* and the accessory genes; subtype D in *env*; and fairly evenly split between subtype A1 and D in *gag*. Three breakpoints were located in *gag*, one in *pol*, one in *tat/rev*, and three in *env*, respectively. In *gag*, a breakpoint was located at either end of p24, suggesting that the entire coding region for the antigen was swapped in the recombination event. Similarly, the third breakpoint was located at the junction of the p7/p1 regions, suggesting that entire coding regions were swapped in the recombination event. The distribution of subtypes in *gag* by protein was A1 (p17, p2, p7) and D (p24, p1, p6).

The single D/A1 breakpoint in *pol* was located approximately 250 bp from the start of the protease; the remainder of the *pol* gene was subtype A1, as were *vif* and *vpr*. The breakpoint located at HXB2 6007 fell in the overlap of *tat* and *rev*; both of these genes were A1/D mosaics. *Vpu* was solely subtype D. Although *env* was largely subtype D, three of the hypervariable regions (V1–V3) were subtype A1.

**Figure 4:**
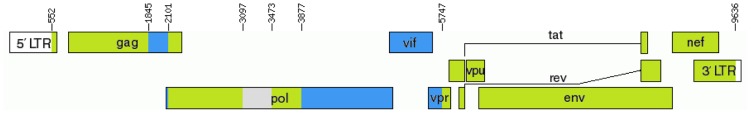
Confirmed structure of the complex recombinant. The confirmed structure of the complex A1/B/D/U recombinant specimen 34567 following maximum likelihood analysis with the CRF50_A1D specimens. CRF50_A1D regions are shown in green and subtype B regions are shown in blue.

The maximum likelihood analysis confirmed that the A1/D isolates clustered monophyletically across the entire genome (Figure 3). The complex isolate 34567 clustered with the A1/D isolates in 7/9 genomic regions; in 2/9 regions this specimen clustered with subtype B reference sequences, confirming that this specimen was a recombinant of the A1/D structure and a subtype B infection (Figures 3 and 4).

### Emergence and distribution of CRF50_A1D

Analysis of the UK HIV-DRD identified a further 67 sequences showing a recombination profile that matched that of CRF50_A1D. The global approximate maximum likelihood trees were built using sequences from East Africa (Kenya, Tanzania, Rwanda, Uganda, Burundi), Central Africa (DRC), Western Africa (Cameroon), Eastern Europe (Latvia, Belarus, Georgia, Russia) and the UK, due to the prevalence of subtypes A and D in these regions. In the approximate maximum likelihood analysis of global alignments of *gag*, *pol and env* gene subtype A and D sequences the CRF50 sequences clustered monophyletically with the East African sequences in both the subtype A and subtype D trees (data not shown). No clustering was observed with subtype A1 or D sequences from the UK. This suggested that CRF0_A1D probably originated in East Africa and was possibly introduced to the UK as a recombinant, rather than emerging from subtype A1 and D strains circulating in the UK.

**Figure 5:**
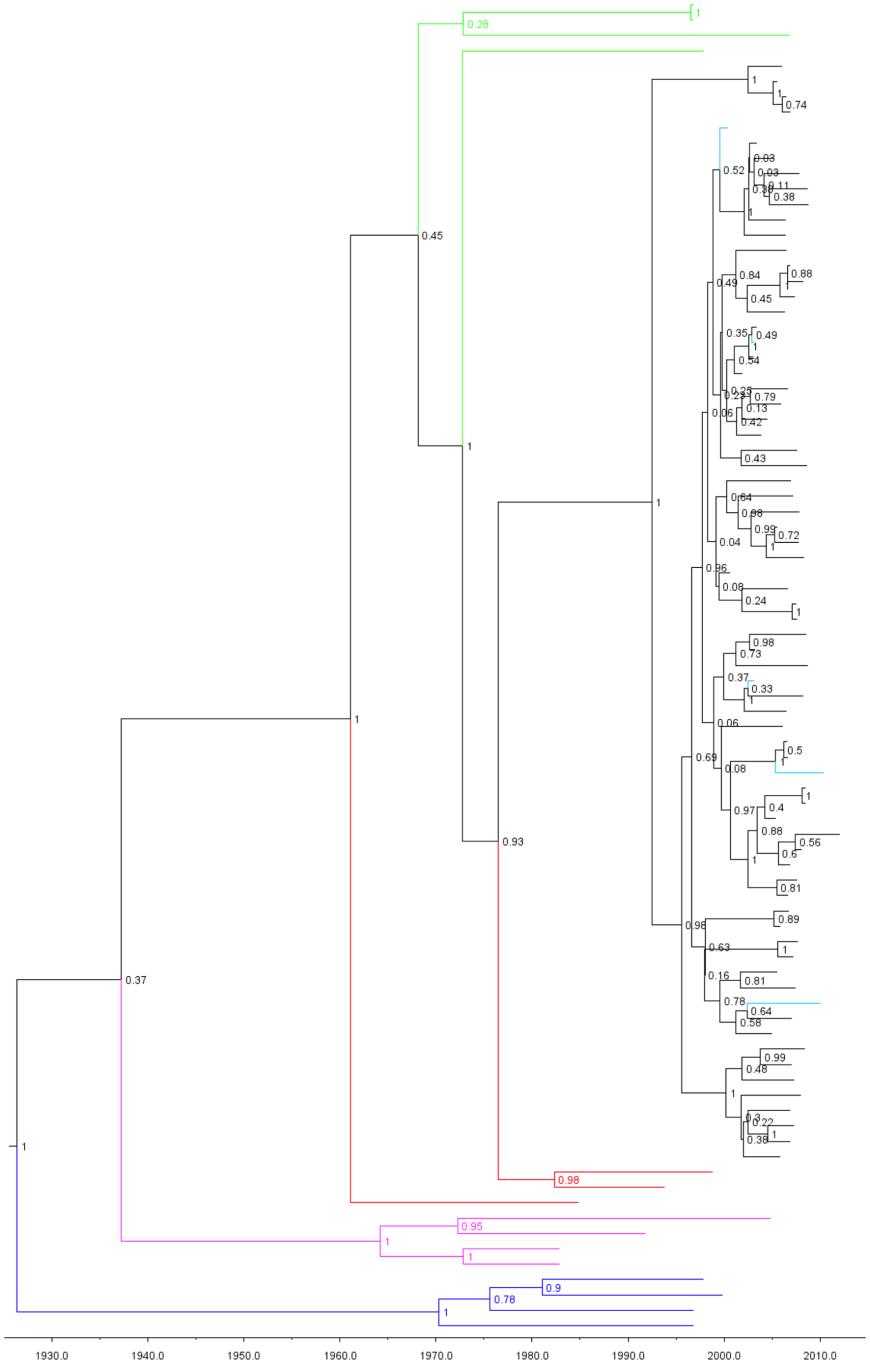
Emergence of CRF50_A1D. Time-scaled analysis of CRF50_A1D *pol* gene sequences using BEAST. Green sequences indicate the closest NCBI BLAST matches to the CRF50 sequences (all subtype A1); red sequences indicate A1 reference sequences; purple sequences indicate subtype D reference sequences. Outgroup subtype C sequences are shown in blue. Node values indicate the posterior probability of each node. The five full-length genomes labeled in turquoise. The emergence of CRF50_A1D in Britain is dated to 1992.

Time-scaled analysis dated the emergence of CRF50_A1D in the UK to 1992 [95% highest posterior density (HPD) 1966–2007; posterior probability 0.9933] (Figure 5). Phylogeographic analysis showed probable emergence in northwest England followed by spread to London and southeast England, with further, limited transmission events in southwest and northeast England and Scotland. Demographic information was available for 51/72 patients infected with CRF50_A1D. Of these, 45 (88.2%) were MSM, 3 (5.9%) were heterosexual males, and 2 (3.9%) were IDUs.

Analysis including the CRF50/B/U unique recombinant form (URF) sequence showed a median divergence date of 2000, indicating an onward recombination event between CRF50_A1D and a subtype B strain. The CRF50_A1D/B/U sequence came from an MSM.

## Discussion

The HIV epidemic in MSM in the UK continues to diversify, creating opportunities for the emergence of novel recombinant forms. By scanning a large national sequence repository, we identified 72 patients who all appeared to be infected with the same novel A1/D recombinant. Near full-length SGS of plasma HIV-1 RNA was performed to characterize the structure of the recombinant. Five patients were found to carry the same A1/D recombinant, which was classified as CRF50_A1D. Based on the recombinant profile, we conclude that CRF50_A1D is the subtype A recombinant that Gifford *et al* hypothesized was circulating among MSM in 2007 [6]. It should be noted that some recombination breakpoints were not identical among the five CRF50_A1D isolates in the jpHMM plots. However, the confidence intervals of the identity estimations and the subsequent analyses indicated that the uncertainties were unlikely to reflect a true alternative recombinant structure. We also found evidence of further genetic evolution of CRF50_A1D through recombination with subtype B, which is the predominant HIV-1 subtype circulating among MSM in the UK. This complex URF was classified as CRF50_A1D/B/U. Crucially, the estimated emergence date of 1992 was both prior to the introduction of highly active antiretroviral therapy and during a period when HIV infections were spreading exponentially in African countries, creating ideal conditions for the creation of novel HIV recombinants which could move into the wider epidemic.

We found a relatively low number of patients infected with this strain within a database that at the time of screening contained 55,556 sequences from 43,002 patients. This relatively modest spread could reflect fitness properties of the CRF. We detected an unusual structure of the *env* gene in this recombinant, in which three out of the five hypervariable regions belonged to subtype A1, whereas the remaining two regions belonged to subtype D. Available data indicate that recombination events in *env* tend to include either the entire gene or at least the entirety of gp120, and this has been related to the functional impact of this protein on viral fitness [15].

We found that CRF50_A1D was related to A1 and D strains of East African origin. A1/D recombinants detected in East Africa have been associated with a fast disease progression, which may limit the number of infections in the community [16]–[19]. It should be noted however that in a sub-analysis, the CD4 cell count slopes before starting ART were similar in MSM infected with subtype B or CRF50_A1D (data not shown). These considerations indicate that CRF50_A1D has potentially interesting phenotypic properties, which would bear further investigation. Further studies are required to indicate whether there is an influence on clinical outcomes or treatment responses.

There are limitations to this study. Our phylogeographic approach dated the emergence of CRF50_A1D in the UK to mid-1992. This study had a limited number of sequences with which to draw this inference. The prevalence of CRF50_A1D was low in the dataset (72/43,002 or 0.17%) with no evidence for rate increase over time. While the UK-DRB comprehensively collects *pol* gene sequences from patients undergoing drug resistance in routine care in the UK, not all HIV centers contribute to the dataset. Furthermore, the database contains only protease and reverse transcriptase sequences and there are similarity between subtype B and subtype D in these genetic regions. Thus it may be proposed that the 72 CRF50_A1D infections identified represent an underestimate. This in turn may potentially bias the estimated date of emergence. Evolutionary analysis of the individual gene using the available full-length sequences and reconstruction of the ancestral subtype A1 and D strains may yield a more precise elucidation of the emergence date and help to determine whether single or multiple introductions occurred in the UK. Furthermore, given that the majority of 72 individuals infected with CRF50_A1D had only partial *pol* sequences available for analysis, it may be postulated that some of these cases may have shown a more complex viral genomic structure if full-length genome analysis had been performed.

The study of novel HIV variants such as CRF50_A1D and the URF CRF50/B/U provides a tool for studying transmission networks and interactions between populations and risk groups, thus producing valuable epidemiological insights [20], [21]. Although in the early years of the HIV epidemic in Western Europe it was rare to find non-B infections in MSM [22], more recent data indicate that non-B infections are not only increasingly important, but are being transmitted indigenously among this population [23]. The use of molecular epidemiological techniques to map these variants can add to our understanding of data gathered using traditional epidemiological means and provides valuable insights into the dynamics of the HIV epidemic that can be used to guide control strategies.

## Supporting Information

Table S1 Sequencing primers used for near full-length HIV-1 single genome sequencing.
(DOCX)
