# Supplementary material for: Novel HIV-1 Recombinants Spreading across Multiple Risk Groups in the United Kingdom: The Identification and Phylogeography of Circulating Recombinant Form (CRF) 50_A1D
Source: PLoS One. 2014 Jan 15;9(1):e83337. doi: 10.1371/journal.pone.0083337 (PMC3893077; doi:10.1371/journal.pone.0083337)
Supplement: Table S1 — Sequencing primers used for near full-length HIV-1 single genome sequencing. (DOCX) [file pone.0083337.s001.docx]

**Table S1.** Sequencing primers used for near full-length HIV-1 single genome sequencing

| Number | Primer Name | Sequence 5’-3’ | HXB2 co-ordinates | Source |
| --- | --- | --- | --- | --- |
|  |  | **Forward primers** |  |  |
| 1 | 2.U5.B4F | AGTAGTGTGTGCCCGTCTGTTGTGTGACTC | 552-581 | CHAVI-MBSC, 2009, unpublished |
| 2 | msf12b(+) | AAATCTCTAGCAGTGGCGCCCGAACAG | 623-649 | Nadai et al., 2008 |
| 3 | KVL066 | TCTCTAGCAGTGGCGCCCGAACAG | 626-649 | Van Laetham et al., 2006 |
| 4 | f2nst(+) | GCGGAGGCTAGAAGGAGAGAGATGG | 769-793 | Nadai et al., 2008 |
| 5 | DD | GTATGGGCAAGCAGGGAGCTAGAA | 892-915 | Nadai et al., 2008 |
| 6 | HH | ATGAGGAAGCTGCAGAATGGG | 1406-1426 | Nadai et al., 2008 |
| 7 | II | ATAATCCACCTATCCCAGTAGGAGAAAT | 1544-1571 | Nadai et al., 2008 |
| 8 | pro5F(+) | AGAAATTGCAGGGCCCCTAGGAA | 1966-2018 | Nadai et al., 2008 |
| 9 | POLCLO1- | GAGAGACAGGCTAATTTTTTAGGGAA | 2071-2096 | Nadai et al., 2008 |
| 10 | pro3F(+) | AGANCAGAGCCAACAGCCCCACCA | 2143-2166 | Nadai et al., 2008 |
| 11 | POLoutF1(+) | CCTCAAATCACTCTTTGGCARCGAC | 2253-2277 | Nadai et al., 2008 |
| 12 | BJPOL1 | ACAGGAGCAGATGATACAGTA | 2328-2348 | Nadai et al., 2008 |
| 13 | POLinF1 | AGGACCTACRCCTGTCAACATAATTGG | 2483-2509 | Nadai et al., 2008 |
| 14 | AZT3 | CCAGGAATGGATGGACCAA | 2589-2607 | Nadai et al., 2008 |
| 15 | SP4S | GGGCCTGAAAATCCATACAATACT | 2700-2723 | Nadai et al., 2008 |
| 16 | AZT9 | TGGATGTGGGTGATGCATA | 2875-2893 | Nadai et al., 2008 |
| 17 | SP5S | GGATTAGATATCAGTACAATGTGC | 2971-2994 | Nadai et al., 2008 |
| 18 | AZT6 | CAATACATGGATGATTTGTATGTAGG | 3093-3118 | Nadai et al., 2008 |
| 19 | POLP | GGATGGGATATGAACTCCATCC | 3235-3256 | Nadai et al., 2008 |
| 20 | DGPOLF7 | GGAATATATTATGACCCATCAAAAGAC | 3495-3521 | Nadai et al., 2008 |
| 21 | POLU | ACTTTCTATGTAGATGGGGCAGC | 3864-3886 | Nadai et al., 2008 |
| 22 | POLI | GAGCAGTTAATAAAAAAGGAA | 4116-4136 | Nadai et al., 2008 |
| 23 | POLJ | GAAGCCATGCATGGACAAGTAGA | 4371-4393 | Nadai et al., 2008 |
| 24 | POLK | ACGGTTAAGGCCGCCTGTTGGTGG | 4602-4625 | Nadai et al., 2008 |
| 25 | POLSEQ2 | CGGGTTTATTACAGGGACAGC | 4899-4919 | Nadai et al., 2008 |
| 26 | VIF1 | GGGTTTATTACAGGGACAGCAGAG | 4900-4923 | CHAVI-MBSC, 2009, unpublished |
| 27 | ACC1 | TTCAGAAGTATACATCCCACTAGG | 5196-5219 | Nadai et al., 2008 |
| 28 | KVL008 | GGTCAKGGRGTCTCCATAGAATGGA | 5284-5308 | Van Laetham et al., 2005 |
| 29 | VIFB | ATATAGCACACAAGTAGACCCT | 5319-5340 | Nadai et al., 2008 |
| 30 | AV317 | TCAAGCAGGACATAAYAAGGTAGG | 5445-5468 | Van Laetham et al., 2005 |
| 31 | VIFC | GAYAAAGCCACCTTTGCCTAGTGTT | 5514-5538 | Nadai et al., 2008 |
| 32 | ENVoutF1 (+) | AGARGAYAGATGGAACAAGCCCCAG | 5550-5574 | Nadai et al., 2008 |
| 33 | ACC5 | TGAAACTTAYGGGGATACTTGG | 5699-5720 | Nadai et al., 2008 |
| 34 | ENVinF1 | TGGAAGCATCCRGGAAGTCAGCCT | 5861-5884 | Nadai et al., 2008 |
| 35 | ENVA | GGCTTAGGCATCTCCTATGGCAGGAAGAA | 5954-5982 | CHAVI-MBSC, 2009, unpublished |
| 36 | ED3 | TTAGGCATCTCCTATGGCAGGAAGAAGCGG | 5957-5986 | Nadai et al., 2008 |
| 37 | GP1205- | AGAGCAGAAGACAGTGGCAATGA | 6206-6228 | Nadai et al., 2008 |
| 38 | Z1F | TGGGTCACAGTCTATTATGGGGTACCT | 6327-6353 | Nadai et al., 2008 |
| 39 | ENVSEQ22 | GTGTACCCACAGACCCCAGCCCACAAG | 6445-6471 | Nadai et al., 2008 |
| 40 | ZFF | GGGATCAAAGCCTAAAGCCATGTGTAA | 6559-6585 | Nadai et al., 2008 |
| 41 | 793SEQ1 | AACACCTCAGTCATTACACAGGCC | 6813-6836 | Nadai et al., 2008 |
| 42 | E16 | CCAATTCCCATACATTATTGTG | 6858-6879 | Nadai et al., 2008 |
| 43 | AV318 | TGCTGYTRAATGGCAGTCTAGCAGA | 7000-7024 | Van Laetham et al., 2005 |
| 44 | E15 | GTAGAAATTAATTGTACAAGACCC | 7098-7121 | Nadai et al., 2008 |
| 45 | OFM54 | TTTAATTGTGGAGGGGAATTTTTCT | 7350-7374 | Nadai et al., 2008 |
| 46 | E13 | ACAAATTATAAACATGTGGCAGG | 7487-7509 | Nadai et al., 2008 |
| 47 | JL109 | GTGAATTATATAAATATAAAGTAG | 7668-7689 | Nadai et al., 2008 |
| 48 | TUG | GTCTGGTATAGTGCAACAGCA | 7859-7879 | Nadai et al., 2008 |
| 49 | ZLF | GGGATAACATGACCTGGATGCAGTGGG | 8092-8118 | Nadai et al., 2008 |
| 50 | JL104 | GGAGGCTTGATAGGTTTAAGAATA | 8292-8315 | Nadai et al., 2008 |
| 51 | JL106 | TTCAGCTACCACCGCTTGAGAGACT | 8520-8544 | Nadai et al., 2008 |
| 52 | NEF7 | TAAGATGGGTGGCAAGTGGTCCAAAA | 8793-8818 | Nadai et al., 2008 |
| 53 | NEF6 | AGCAGCAGATGGGGTGGGAGCAG | 8871-8893 | Nadai et al., 2008 |
| 54 | LTR2 | TTTGGATGGTGCTACAAGCTA | 9211-9231 | Designed using Primer3 |
|  |  | **Reverse primers** |  |  |
| 55 | JL19 | CTTCTATTACTTTTACCCATGC | 1249-1270 | Nadai et al., 2008 |
| 56 | JL17 | CATTCTGCAGCTTCCTCATTGAT | 1402-1424 | Nadai et al., 2008 |
| Number | Primer Name | Sequence 5’-3’ | HXB2 co-ordinates | Source |
| 57 | SP2AS | GGTGGGGCTGTTGGCTCTG | 2147-2165 | Nadai et al., 2008 |
| 58 | SP3AS | CCTCCAATTCCCCCTATCATTTTTGG | 2382-2407 | Nadai et al., 2008 |
| 59 | KVL067 | GGCCATTGTTTAACYTTTGGDCCATCC | 2597-2623 | Van Laetham et al., 2006 |
| 60 | SP4AS | AGTATTGTATGGATTTTCAGGCCC | 2700-2723 | Nadai et al., 2008 |
| 61 | KVL065 | TCCTAATTGAACYTCCCARAARTCYTGAGTTC | 2797-2828 | Van Laetham et al. |
| 62 | POLC- | CTAGGTATGGTAAATGCAGTATA | 2928-2950 | Nadai et al., 2008 |
| 63 | AZT10 | CCTACATACAAATCATCCATGTATTG | 3093-3118 | Nadai et al., 2008 |
| 64 | AZT5 | TCAGATCCTACATACAAATCATCCATGTATTG | 3093-3124 | Nadai et al., 2008 |
| 65 | AZT4 | TATAGGCTGTACTGTCCATTT | 3261-3281 | Nadai et al., 2008 |
| 66 | POLEE- | TGTATGTCATTGACAGTCCAGCTG | 3299-3322 | Nadai et al., 2008 |
| 67 | proRT | TTTCCCCACTAACTTCTGTATGTCATTGACA | 3308-3338 | Nadai et al., 2008 |
| 68 | RT3473R (-) | GAATCTCTCTGTTTTCTGCCAGTTC | 3453-3477 | Nadai et al., 2008 |
| 69 | POLSEQ3 | GATATGWCCACTGGTCTTGCCC | 3546-3567 | Nadai et al., 2008 |
| 70 | DGPOL3R | GTATTGACAAACTCCCAGTCAGGAAT | 3780-3805 | Nadai et al., 2008 |
| 71 | POLI- | TTTGTGTGCTGGTACCCATGCCAG | 4146-4169 | Nadai et al., 2008 |
| 72 | POLT- | GCAGTCTACTTGTCCATGCATGGC | 4374-4397 | Nadai et al., 2008 |
| 73 | SP1AS | GGATGAATACTGCCATTTGTACTGC | 4752-4776 | Nadai et al., 2008 |
| 74 | DGPOL2R | CACTATTGTCTTGTATTACTAC | 4974-4995 | Nadai et al., 2008 |
| 75 | ACC2 | AGGGTCTACTTGTGTGYTATAT | 5319-5340 | Nadai et al., 2008 |
| 76 | ACC6 | GCTTGTTCCATCTRTCYTCTGTYAG | 5545-5569 | Nadai et al., 2008 |
| 77 | ACC4 | CCAAGTATCCCCRTAAGTTTCA | 5699-5720 | Nadai et al., 2008 |
| 78 | ACC8R | TCTCCGCTTCTTCCTGCCATAG | 5968-5989 | Nadai et al., 2008 |
| 79 | VIF-VPUinR1 | CTCTCATTGCCACTGTCTTCTGCTC | 6207-6231 | Nadai et al., 2008 |
| 80 | ES33 | CATTGCCACTGTCTTCTGCTC | 6207-6227 | Nadai et al., 2008 |
| 81 | VIF-VPUoutR1 (-) | GGTACCCCATAATAGACTGTRACCCACAA | 6324-6352 | Nadai et al., 2008 |
| 82 | JL99 | TTTAGCATCTGATGCACAAAATAG | 6378-6401 | Nadai et al., 2008 |
| 83 | AENVSEQ4 | CAAGCTTGTGTAATGGCTGAGG | 6817-6838 | Nadai et al., 2008 |
| 84 | TUE3 | TCCTTCTGCTAGACTGCCATTTA | 7006-7028 | Nadai et al., 2008 |
| 85 | JL98 | AGAAAAATTCCCCTCCACAATTAA | 7351-7374 | Nadai et al., 2008 |
| 86 | JL102 | GATGGGAGGGGCATACAT | 7509-7524 | Nadai et al., 2008 |
| 87 | EDS8 | CACTTCTCCAATTGTCCCTCA | 7648-7668 | Nadai et al., 2008 |
| Number | Primer Name | Sequence 5’-3’ | HXB2 co-ordinates | Source |
| 88 | AV323 | CTGCTCCYAAGAACCCAA | 7783-7800 | Van Laetham et al., 2005 |
| 89 | TUH | GCCCCAGACTGTGAGTTGCAACAGATG | 7914-7940 | Nadai et al., 2008 |
| 90 | FM116 | CAGAGATTTATTACTCCAACTA | 8060-8081 | Nadai et al., 2008 |
| 91 | ENVSEQ6 | CCTGCCTAACTCTATTCAC | 8337-8355 | Nadai et al., 2008 |
| 92 | E8 | CTCTCTCTCCACCTTCTTCTTC | 8424-8445 | Nadai et al., 2008 |
| 93 | JL71 | TTTTGACCACTTGCCACCCAT | 8797-8817 | Nadai et al., 2008 |
| 94 | AV319 | GCTSCCTTRTAAGTCATTGGTCT | 9025-9047 | Van Laetham et al., 2005 |
| 95 | JL89 | TCCAGTCCCCCCTTTTCTTTTAAAAA | 9064-9089 | Nadai et al., 2008 |
| 96 | KVL009 | GCCAATCAGGGAAGWAGCCTTGTGT | 9145-9169 | Van Laetham et al., 2005 |
| 97 | nefyn05 (-) | GTGTGTAGTTCTGCCAATCAGGGAA | 9157-9181 | Nadai et al., 2008 |
| 98 | UNINEF 7’ (-) | GCACTCAAGGCAAGCTTTATTGAGGCTT | 9605-9632 | Nadai et al., 2008 |
| 99 | OFM19 | GCACTCAAGGCAAGCTTTATTGAGGCTTA | 9604-9632 | CHAVI-MBSC, 2009, unpublished |
| 100 | 2.R3.B6R | TGAAGCACTCAAGGCAAGCTTTATTGAGGC | 9607-9636 | CHAVI-MBSC, 2009, unpublished |
